# A raster-based spatial clustering method with robustness to spatial outliers

**DOI:** 10.1038/s41598-024-53066-4

**Published:** 2024-02-19

**Authors:** Haoyu Wang, Changqing Song, Jinfeng Wang, Peichao Gao

**Affiliations:** 1 Faculty of Geographical Science, Beijing Normal University, Beijing, 100875 China; 2 Institute of Geographic Sciences and Natural Resources Research, Chinese Academy of Sciences, Beijing, 100101 China; 3 State Key Laboratory of Earth Surface Processes and Resource Ecology, Beijing Normal University, Beijing, 100875 China

**Keywords:** Computational science, Statistics

## Abstract

Spatial clustering is an essential method for the comprehensive understanding of a region. Spatial clustering divides all spatial units into different clusters. The attributes of each cluster of the spatial units are similar, and simultaneously, they are as continuous as spatially possible. In spatial clustering, the handling of spatial outliers is important. It is necessary to improve spatial integration so that each cluster is connected as much as possible, while protecting spatial outliers can help avoid the excessive masking of attribute differences This paper proposes a new spatial clustering method for raster data robust to spatial outliers. The method employs a sliding window to scan the entire region to determine spatial outliers. Additionally, a mechanism based on the range and standard deviation of the spatial units in each window is designed to judge whether the spatial integration should be further improved or the spatial outliers should be protected. To demonstrate the usefulness of the proposed method, we applied it in two case study areas, namely, Changping District and Pinggu District in Beijing. The results show that the proposed method can retain the spatial outliers while ensuring that the clusters are roughly contiguous. This method can be used as a simple but powerful and easy-to-interpret alternative to existing geographical spatial clustering methods.

## Introduction

Spatial clustering, also known as regionalization, is a traditional research topic in geography. Spatial clustering is when a set of spatial objects are grouped into several subsets, which are internally similar and as spatially contiguous as possible. These subsets are called clusters (or regions). Spatial clustering reveals the common processes and characteristics within the clusters and the differences between the clusters^1^. The new clusters have a more comprehensive geographic extent than the original spatial objects^2^, making clusters the foundation of further integrated research^3^. Spatial clustering has been used in many disciplines in geography^4–7^. Based on the spatial differentiation of ecosystem functions and ecological environment, ecological function zoning provides a scientific foundation for comprehensive ecological management policies^8–10^. Based on DEM and other morphometric attributes, geomorphometric regionalization plays a vital role in understanding the Earth’s surface processes, natural hazards and landscape evolutions^11,12^.

Spatial clustering encounters a significant challenge in adapting standard clustering techniques like K-means to capture spatial relationships^13^. To address the adaptation process, a careful balance between capturing spatial relationships and preserving attribute similarity is required. On the one hand, according to the first law of geography, nearby things are more closely related^14^, which makes nearby spatial units tend to be clustered into one cluster in spatial clustering. However, this process can result in the loss of similarity within the cluster. On the other hand, standard clustering methods disregard spatial relationships and focus solely on maximizing dissimilarity between clusters and maximizing similarity within clusters, which can lead to fragmented spatial clustering results.

To achieve a reasonable tradeoff between spatial and attribute relationships, the handling of spatial outliers is crucial. Spatial outliers refer to spatial units that are quite inconsistent with the attribute values of the surrounding units^15^. In practical geographical datasets, these spatial outliers can originate from various factors, such as errors in data collection, natural spatial variations, or the influences of human activities. These spatial outliers possess the capacity to severely disrupt the functionality of conventional clustering algorithms, resulting in clustering outcomes that lack the precision and reliability required for credible geospatial analysis. Specifically, if the spatial outliers are clustered into the same cluster with the surrounding units based on geographical location, the substantial attribute differences can severely damage the similarity within the cluster. Hence, enhancing the robustness of spatial clustering methods against spatial outliers is of paramount importance. Improving the robustness to spatial outliers ensures that spatial clustering is less susceptible to being significantly affected by them, regardless of how the tradeoff between spatial and attribute relationships is managed. Nevertheless, despite the availability of numerous spatial outlier detection algorithms^16^, there is currently a shortage of spatial clustering algorithms designed to enhance robustness against spatial outliers.

Raster data essentially represent a region as a matrix of grids, and each grid stores the value of a spatial attribute^17^. The location of a cell is defined by its row and column numbers. The spatial clustering of raster data divides each grid into different clusters according to its attribute value and location. The attributes of the same cluster are similar, and the spatial position is contiguous. A great deal of research in geography employs raster data, especially when expressing information that varies continuously in space. This information is often challenging to segment and represented as vector data, such as temperature, elevation, or spectral data. In addition, advances in remote sensing technology have enriched raster data. Satellite images or aerial photos are all raster data.

This paper proposes a spatial clustering of raster data that is robust to spatial outliers. It is easy to understand and convenient to implement. This method explores the range and standard deviation of the attribute values of each locality through a sliding window. Based on this, the classification results of the raster data are adapted to improve spatial integration while protecting spatial outliers. In addition, we designed a comparison experiment for spatial clustering methods to compare the clustering effects of different types of spatial clustering methods.

## Related works

### Applications of spatial clustering

Spatial Clustering serves two primary applications^13^. First, it plays a crucial role in data preprocessing. It serves as an automated, unsupervised method for data organization. This enhances the efficiency of search or query algorithms and contributes to the overall system’s performance. Secondly, cluster analysis is employed in exploratory tasks, enabling knowledge discovery and stimulating the generation of new hypotheses by identifying underlying patterns. In the context of data preprocessing, spatial clustering can help organize geographical data, making it more convenient for further data mining operations^18^. Some studies utilize spatial clustering for image feature matching^19,20^. Spatial clustering is also a significant method for outlier detection^21^. Additionally, research has shown that spatial clustering can enhance the efficiency of spatial query processing^22,23^.

In the domain of exploratory research, spatial clustering finds extensive applications across various fields. For instance, in climatology, Wang et al.^24^. employed spatial clustering to analyze urban signatures in Precipitation Extremes over Mainland China. In urban studies, Yu et al.^25^ used nighttime light data for spatial clustering to delineate urban spatial clusters and assess urban landscape patterns. In public health, researchers employ spatial clustering to analyze factors influencing the spread of infectious diseases^26,27^. In agriculture, Gao et al^28^. utilized spatial clustering for the analysis of agricultural soil data. In the field of remote sensing, spatial clustering serves as a crucial tool for processing hyperspectral images^29,30^.

### Existing methods for clustering spatial data

Spatial clustering methods cluster spatial units with similar attributes while imposing constraints of being spatially contiguous^31^. There are two ways to impose constraints, imposing strict constraints or non-strict constraints^32^. A strict constraint means that the spatial units of the same cluster must be contiguous. Non-strict constraints require that only most, but not all, spatial units in a cluster be contiguous. The different clustering results of the two types of constraints are shown in Supplementary Figure S1.

The strict spatially constrained clustering methods require that all of the spatial units of a cluster be contiguous. These spatial clustering methods are also referred to as regionalization methods. These methods can be divided into two types^33^. The first type of algorithm achieves spatial integration by postprocessing the results of standard clustering or maximizing regional compactness. For example, ^34^ regarded spatially contiguous parts of the clustering result as clusters. The second type of algorithm incorporates spatial integration into the clustering process as an explicit constraint. For example, adapted hierarchical clustering aggregates two contiguous and most similar clusters at each step^35^.

Strict spatially constrained clustering methods have disadvantages. First, some spatial units that are very similar but far away will be divided into different clusters^32^. Second, there are some contiguous but not remarkably similar units in one cluster. Moreover, this type of method fails when it is allowed to sacrifice spatial integration to some extent (e.g., allowing enclaves) to improve attribute similarity. Non-strict spatially constrained clustering methods can avoid these disadvantages to a certain extent.

The non-strict spatially constrained clustering methods trade off between spatial integration and attribute similarity^36^ divided spatial clustering methods into four categories: strict spatially constrained clustering and the other three are all non-strictly constrained clustering^37^ held the same opinion. That is, non-strict spatially constrained clustering methods can be divided into three categories. The first category considers the geographical coordinates as additional attributes. The trade-off between attribute similarity and locational similarity is reflected by adjusting the weights of the geographical coordinates^38,39^. For example^40^, proposed a bicriterion median clustering problem by adding geographic coordinates to median clustering for spatial units with a single attribute. The second category modifies the dissimilarity measure between different spatial units of the nonspatial clustering methods. The modified dissimilarity measure combines attribute dissimilarity and locational dissimilarity^41^ first proposed a spatial clustering method for this category, and the modified dissimilarity measure was applied to K-means clustering. Some other nonspatial clustering methods have been modified to this category more recently^32^ proposed a hierarchical clustering algorithm including spatial constraints and implemented it with the R package ClustGeo^42^ proposed an improved version ClustGeo, Bootstrap ClustGeo. All the methods employed a weighted sum of the attribute and locational dissimilarity. The third category is based on statistical models^36^. Ambroise et al.^36^ proposed a spatial clustering method based on Markov random field, which was first used for image segmentation^43^ proposed a software SpaCEM3 in which spatial clustering using Markov models is implemented. This software applied the model for gene clustering based on the Markov models proposed by^44^.

These categories of methods have limitations in handling spatial outliers. For the first two categories, spatial integration and attribute similarity are controlled by location weights (namely, the weights of geographical coordinates and locational dissimilarity). Suppose the weight of the location is too large. In that case, the spatial outliers will be clustered with closer spatial units, resulting in excellent attribute dissimilarity of this cluster. If the weight of the location is too small, although the spatial outliers can be correctly delineated, the resulting clusters are spatially scattered and have poor spatial integration. The third category of methods requires the spatial units involved in clustering to satisfy certain statistical assumptions, such as the independence assumption^37^. For geographic problems, these assumptions cannot often be satisfied, and the methods will perform poorly, so this type of category will not be considered in this paper. To reasonably delineate spatial outliers, this paper proposes a new non-strict spatially constrained clustering method for univariate spatial data (spatial data with a single attribute).

## A new method for clustering raster data

### Overview of the strategy

**Figure 1:**
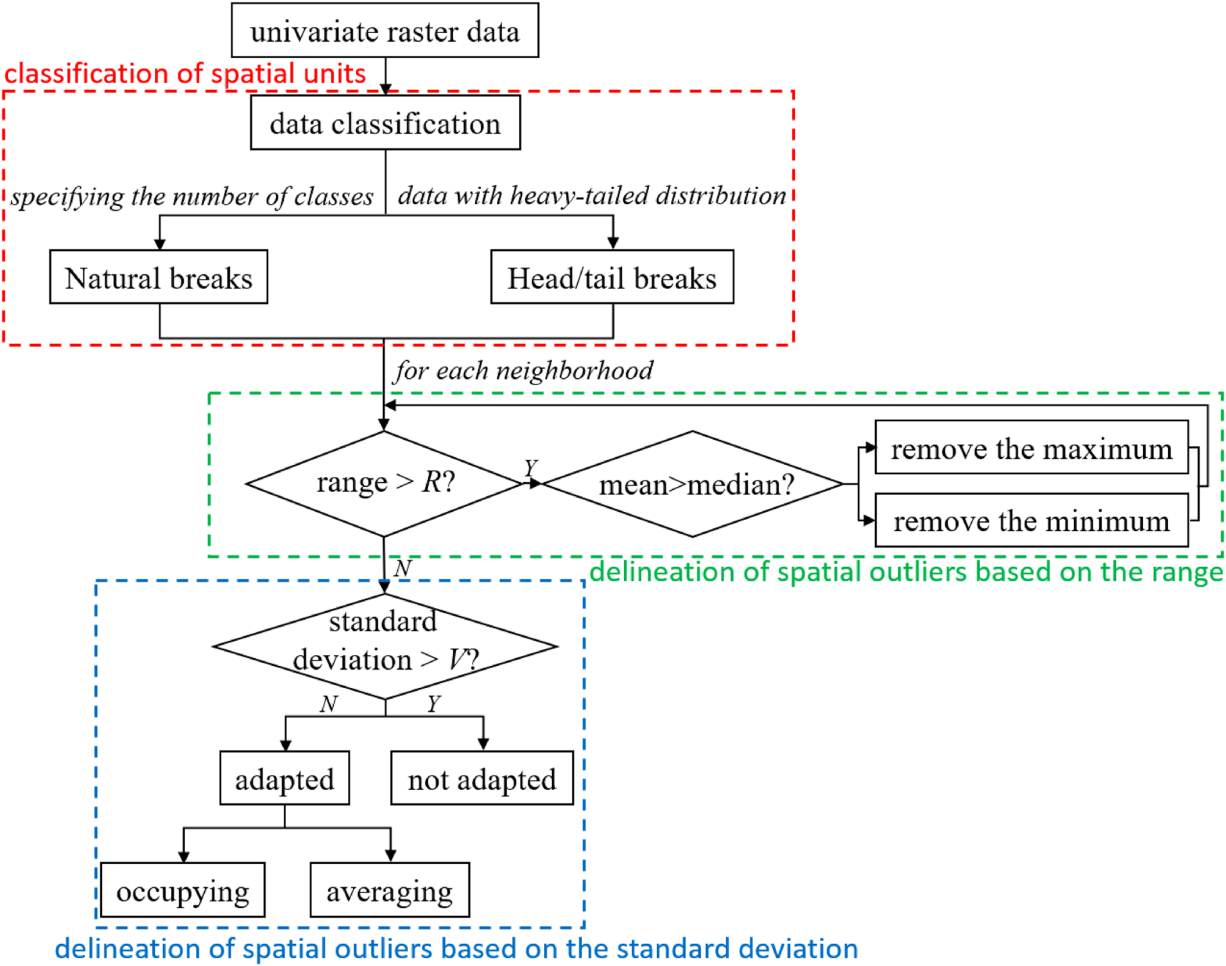
The framework of the proposed method.

Our method is based on two fundamental principles. The first is the contiguousness principle. Spatial units of the same cluster should be connected as much as possible. The second is the reservation principle. The difference should be preserved when the spatial unit differs too much from the surrounding attributes. This principle makes it possible to characterize spatial outliers in contiguous areas. The new method proposed in this paper performs spatial clustering by improving nonspatial clustering. At present, for the spatial clustering of raster data, the method of data classification is mainly used, and the raster is classified according to an attribute value^11,45–47^. We use data classification schemes for nonspatial clustering for univariate data. Then the method adapts the nonspatial clustering results of some spatial units to improve spatial integration. As stated before, the spatial outliers deviate considerably from the values of the attributes of their spatial neighborhood. Hence, it is important to define how ‘considerable deviation’ is in the method^48^. In statistics, range and standard deviation are two commonly used measures of difference. So, the range and standard deviation of the values of the attributes of the spatial unit and its neighbors are used in the method to portray the spatial outliers. In general, the strategy of the proposed method consists of three steps. The first step is to classify spatial units based on attribute values to achieve nonspatial clustering. The second step is to delineate the spatial outliers using the range. The third step is to delineate the spatial outliers using the standard deviation. The framework is shown in Fig. 1.

In the proposed method, the principle of spatial outlier protection ensures the attribute similarity within the cluster, while the principle of contiguousness improves the spatial integration of the clusters of units other than spatial outliers. This is the way the proposed method as a non-strictly constrained clustering tradeoffs spatial integration and attribute similarity. Based on this, the proposed method can reasonably cluster spatial outliers under various degrees of spatial and attribute trade-offs, i.e., it is robust to spatial outliers.

### Classification of spatial units

In our method, two data classification schemes, the natural breaks and head/tail breaks, are provided to perform nonspatial clustering. The natural breaks scheme follows the clustering principle, minimizing the variance within the class while maximizing the variance between classes. At the same time, the number of classes must be specified in advance. In contrast, the head/tail breaks can adaptively determine the number of classes. Nevertheless, this scheme is specifically designed for data with heavy-tailed distributions. Users can choose an appropriate data classification scheme according to their needs. For univariate raster data, each grid is taken as a spatial unit, and the attribute values of all the spatial units are classified.

The natural breaks scheme is the most popular data classification scheme and has been widely used in geography^49,50^. The natural breaks scheme classifies data into several classes in light of the breaks or gaps in the data^51^, which is done by maximizing the between-class variance and minimizing the within-class variance^52^. This data classification scheme follows the principle of data clustering^53^. It should be noted that when performing natural breaks, the user needs to specify the number of classes.

The head/tail breaks scheme is a new data classification method^49,51^. It is used to classify data with a heavy-tailed distribution. In a heavy-tailed distribution, the histogram of the data values is right-skewed. A majority of data values are small, and a minority of data values are large, which can also be stated as ’there are far more small things than large ones’^51^. Heavy-tailed distributions have been detected in many social and natural phenomena. The head/tail breaks scheme first divides the data into two parts based on the mean. The part above the mean is called the head, which is the minority. The part below the average is called the tail, which is the majority. The division is continued for the head iteratively until the head part is no longer heavy-tailed^54^. Unlike the natural breaks, the head/tail breaks scheme does not require the user to specify the number of classes.

### Delineation of spatial outliers based on the range

**Figure 2:**
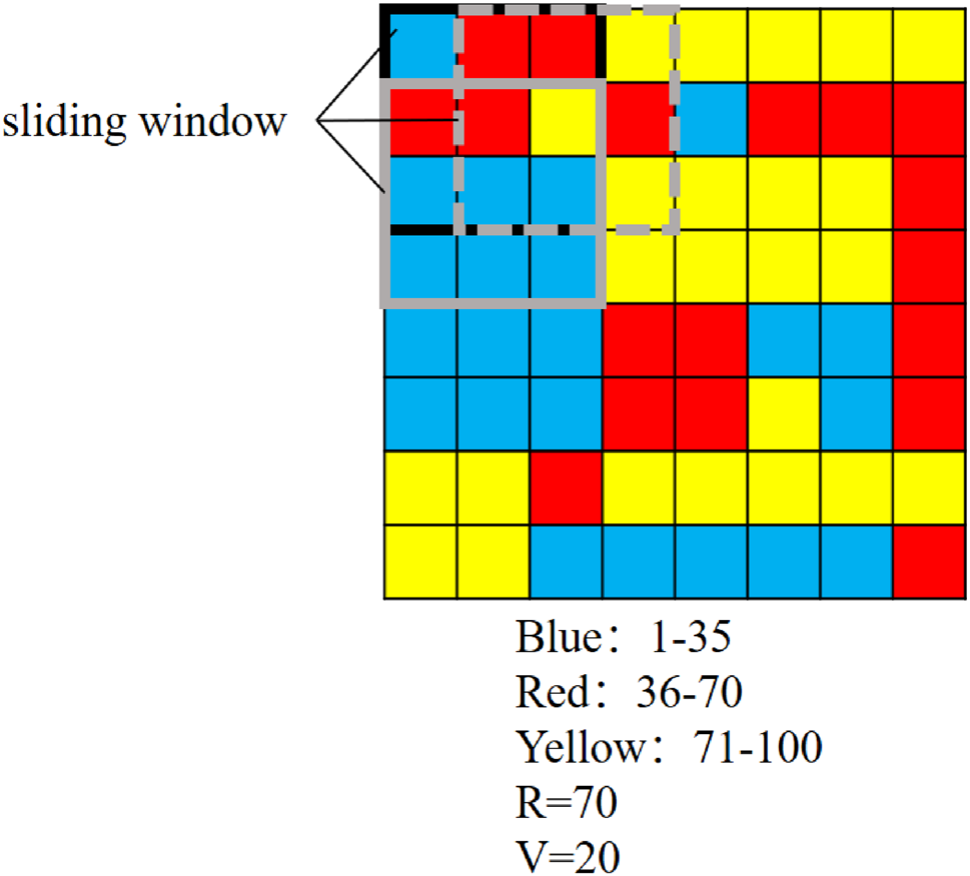
An example of a sliding window.

Before the following two steps, the shape and size of the neighbourhood need to be determined. Since, in raster data, all the spatial units are uniform in size and neatly arranged, a fixed-size square is selected as the neighbourhood. If a square with side length n is set as a neighbourhood, each group of n rows and n columns of the spatial units (grids) forms a neighbourhood. Then, the second and third steps can be performed for each neighbourhood. Users decide the fixed side length. The fixed-size square slides across the whole research area so that the classes of all the spatial units can be adapted. We also call the fixed-size square a ‘sliding window’, and the sliding step is one grid. An example of a sliding window is shown in Fig. 2. The whole figure represents the classification of the research area, and the different colours represent different classes. The side length of the sliding window is three in this example, as the black box is shown in the top left corner of the area. Each area consisting of grids of three rows and three columns is a sliding window, such as the grey dashed box and the solid grey box.

**Figure 3:**
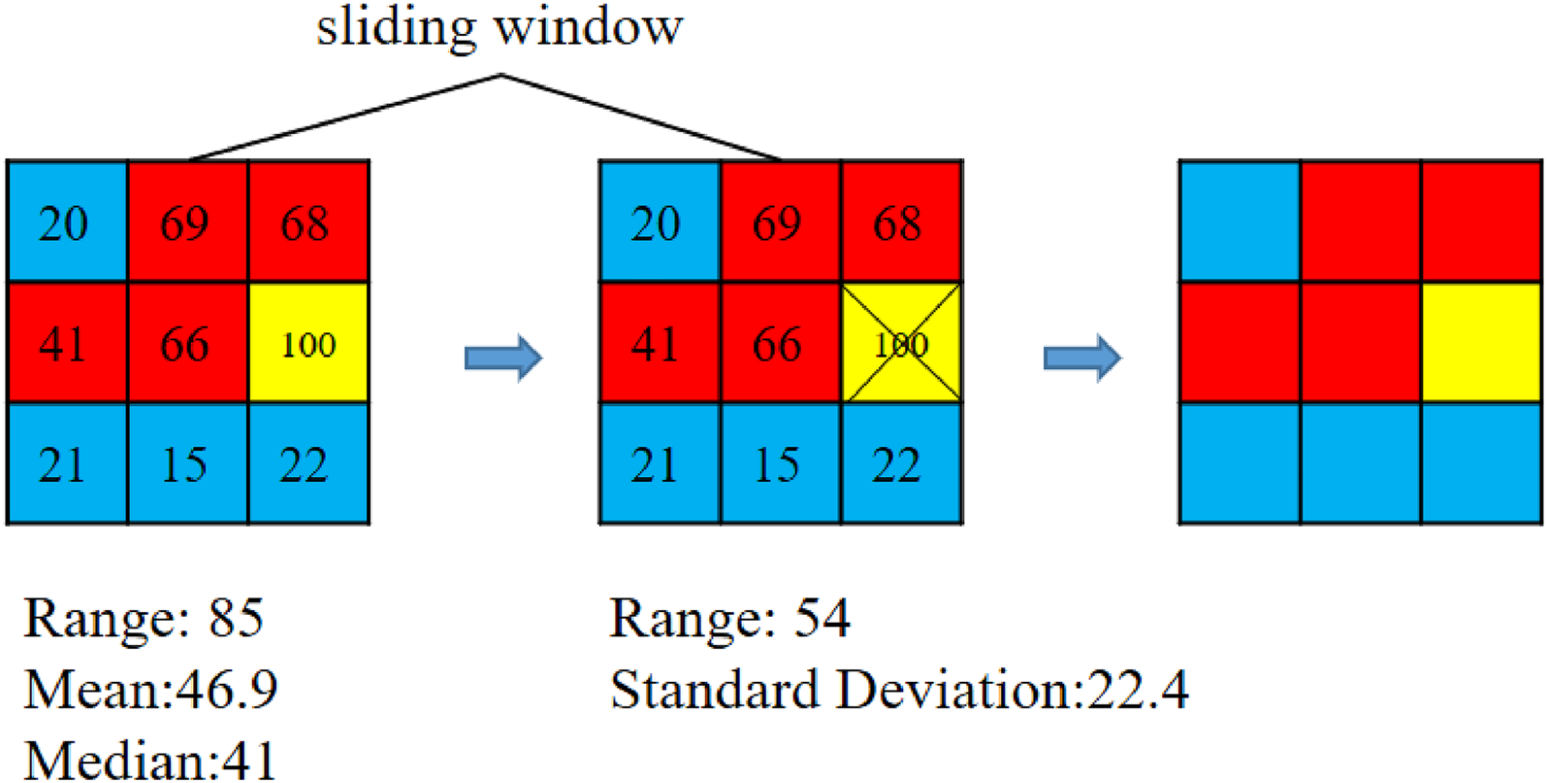
An example of delineation of spatial outliers based on the range.

**Figure 4:**
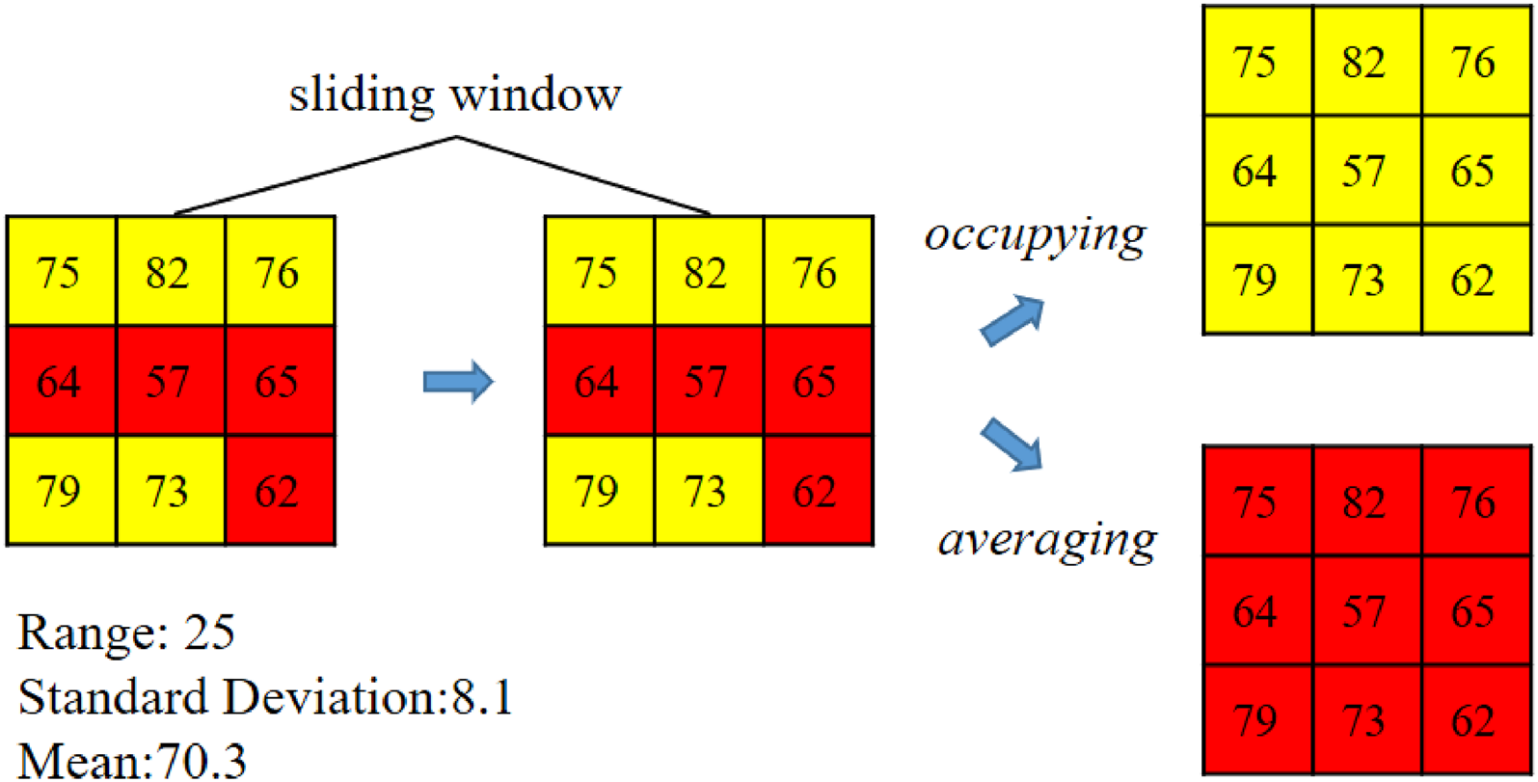
An example of delineation of spatial outliers based on the standard deviation.

Spatial outliers are delineated and preserved based on the range of the attribute values of the spatial units in the sliding window. If the range is too large, it is considered that there are spatial outliers in the sliding window; otherwise, there are no spatial outliers. Spatial outliers will be preserved. The criteria for judging whether the range is large or not is through the threshold R given by the user. If the range is greater than R, the grid with an extreme attribute value, which means either a maximum or minimum, will be removed from the sliding window. If the mean attribute value in the sliding window is greater than the median, the grid with the maximum attribute value should be removed; otherwise, the grid with the minimum value should be removed . Then, the above process is repeated until the range is less than R. The grids removed from the sliding window are spatial outliers whose classes will be preserved; they will not be adapted anymore. The relationship between the number of grids removed from the window and the range threshold is shown in Supplementary Figure S2. Examples of the preservation of spatial outliers are shown in the left half of Fig. 3 and Fig. 4. Two sliding windows are shown in the left subfigures with black borders in Figs. 3 and 4, and the threshold R was set to 70. The numbers in the grids represent the attribute values. The range in the window on the left in Fig. 3 is 85, which is greater than 70. Therefore, there will be a grid removed from the window. Since the mean of the window is greater than the median, the grid with the largest attribute value, 100, should be removed. Then, the range of the remaining attribute values is 54 less than 70, so the process ends. While the range of the window in Fig. 4 is 25, which is less than 70, no grids will be removed. The results are shown in the middle subfigures.

### Delineation of spatial outliers based on the standard deviation

Before introducing this step, it is necessary to clarify the concepts. Since we chose data classification schemes as nonspatial clustering methods, to avoid confusion, the results of the data classification (i.e., nonspatial clustering) are called *classes*, and the adapted results (i.e., the results of spatial clustering) are called *clusters*.

Spatial outliers are further delineated and preserved based on standard deviation. Similarly, a threshold (denoted as V) is needed to determine whether the standard deviation is large. If the standard deviation of the attribute values in the sliding window is greater than V, which means the attribute values fluctuate, the areas in the window are also considered spatial outliers. Grids in the window cannot be adapted at this point. If the standard deviation is less than V, the grids in the window will be adapted to the same cluster to improve the spatial integration. There are two adaptation mechanisms. The first one is called ‘*occupying*’. Grids will turn into the class most resident in the window in this mechanism. The other is called ‘*averaging*’. In this mechanism, grids will turn into the class according to the mean of the attribute values in the window. This process is illustrated in the right half of Figs. 3 and 4. The threshold V is set to 20. The standard deviation in the sliding window in the middle part of Fig. 3 is 22.4, so these grids cannot be adapted. Since the standard deviation in the sliding window in the middle part of Fig. 4 is 8.1, this sliding window should be adapted. The most resident class is yellow by the occupying mechanism, so the whole window turns yellow. By the averaging mechanism, the mean of the attribute values in the modified window is 70.3, so the corresponding class is red. The results are shown in the right parts of Figs. 3 and 4. The whole process can be likened to an ‘infection’ and ‘quarantine’. The spatial units that are quite different from the surrounding area are ‘quarantined’ and are not affected by the outside. The rest of the spatial units will be ‘infected’ with the same type.

## Experimental design

To evaluate the robustness to spatial outliers of the proposed method, we designed two experiments on two sets of raster data. Then, the results obtained by the proposed method are compared with the baseline methods.

### Experimental data

**Figure 5:**
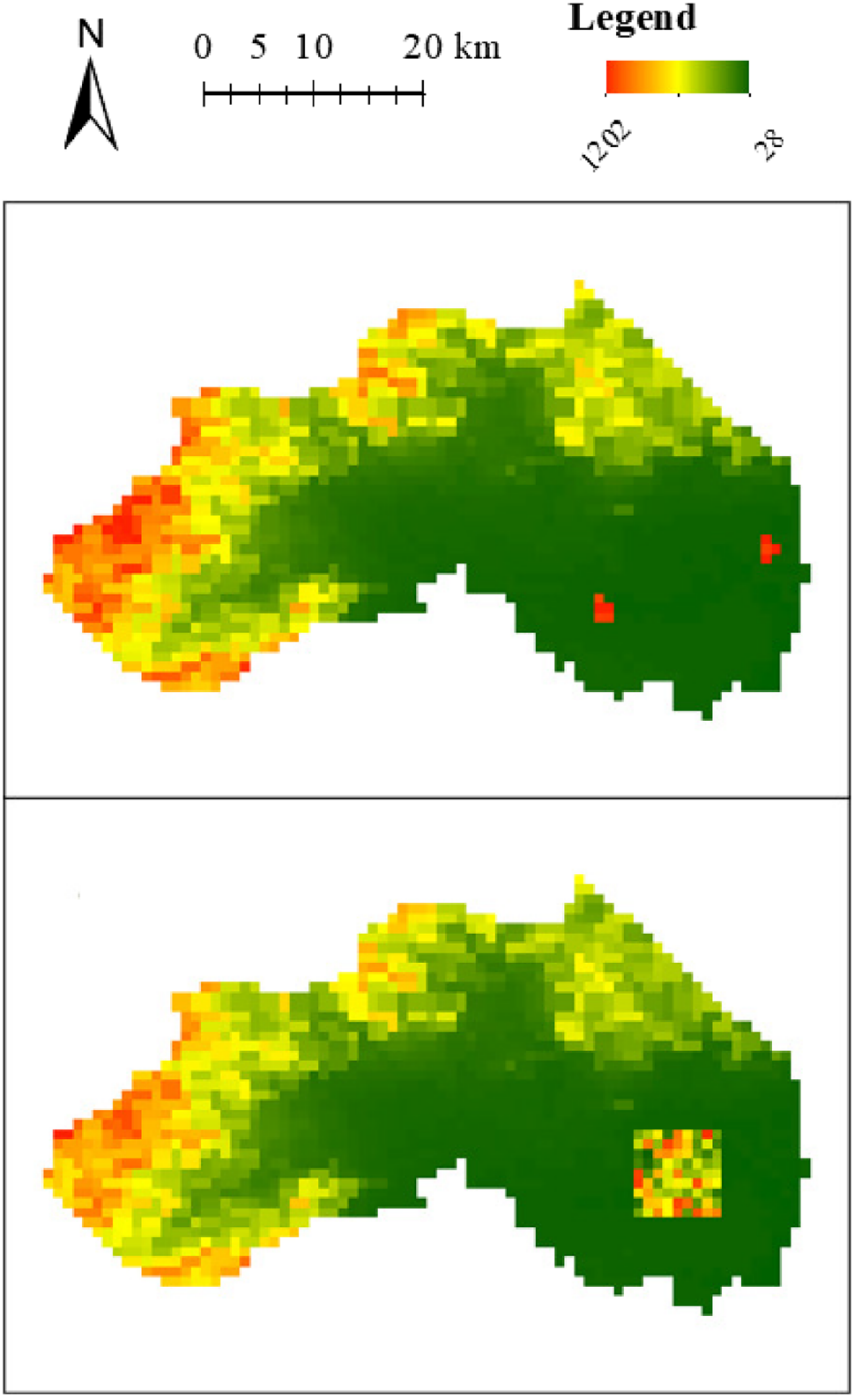
Extreme areas (top) and volatility area (bottom) in Changping District.

**Figure 6:**
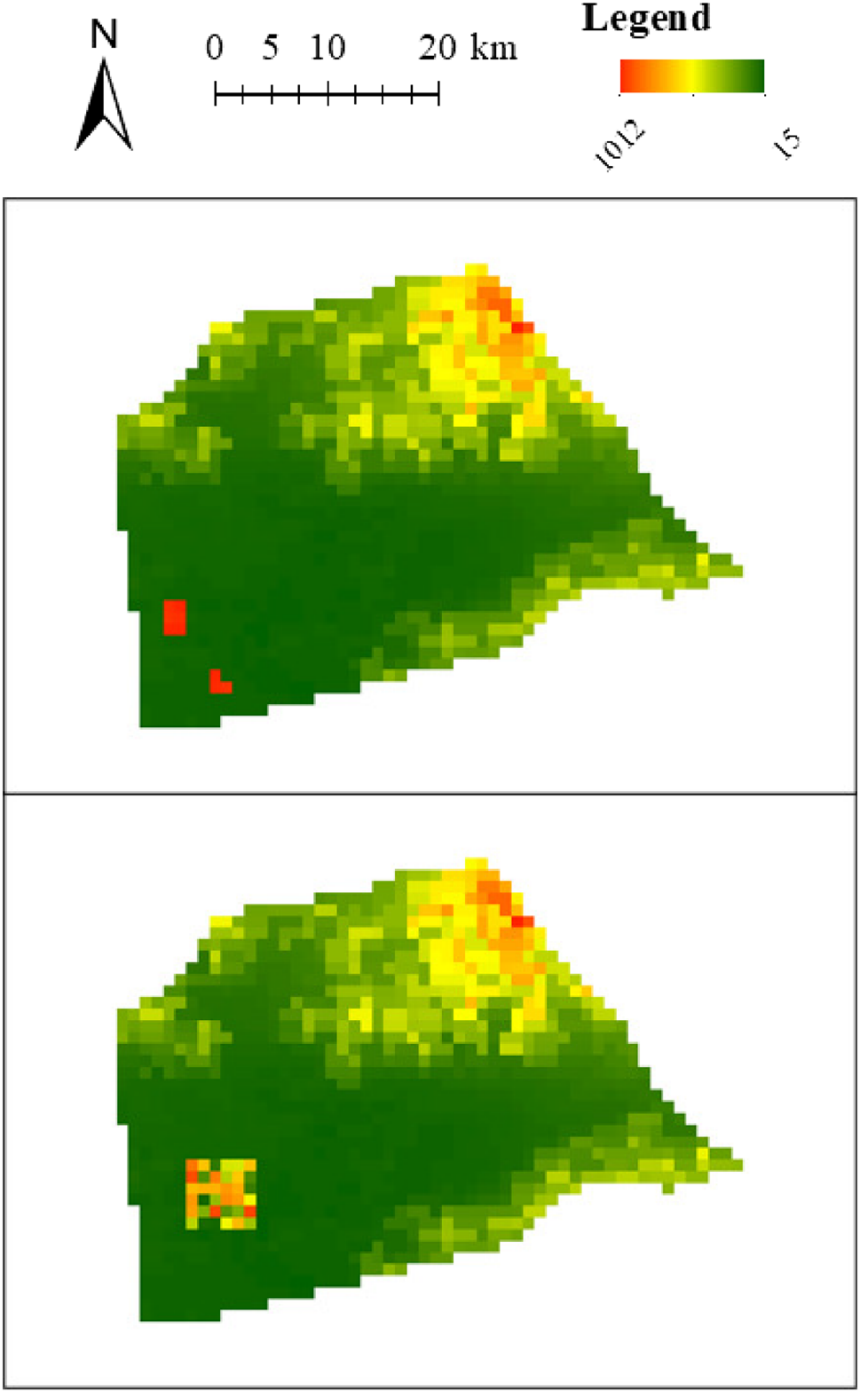
Extreme areas (top) and volatility area (bottom) in Pinggu District.

We selected the DEM data of two districts in Beijing, Changping District and Pinggu District, to evaluate the algorithm; each grid has an attribute value of DEM. The DEM data in Pinggu District are heavy-tailed distributions, while those in Changping District are not heavy-tailed distributions. It can be noted that both districts have large areas of low value (southeastern Changping District and southwestern Pinggu District). To test the robustness of the methods to spatial outliers, we used the method to cluster two representative types of outlier areas that are difficult to delineatee, extreme areas and volatility areas, which refer to areas with large and fluctuating differences in attribute values compared to surroundings, respectively. To test the clustering of extreme and volatility areas, the attribute values of several grids in the low-value region were changed. The attribute values of several grids were changed from low values to high values to construct the extreme areas. The attribute values of some grids were changed to random values to construct the volatility areas. The maps after changing the attribute values of the two districts are shown in Figs. 5 and 6.

### Baseline method selection

We compared the proposed method with two baseline methods. The first, spatial K-means (SKM for short), is commonly used among the first category of non-strict spatially constrained clustering methods. The second, a Ward-like hierarchical clustering algorithm including spatial/geographical constraints (WHS for short), is a recently proposed method in the second category of non-strict spatially constrained clustering methods. Spatial K-means is an adaptation of K-means clustering that treats the coordinates of each spatial unit as two attributes, and the weights of the coordinates can be selected. For example, if the weight of the coordinates is set to 0.2, the weights of the x and y coordinates are 0.1, and the weight of the nonspatial attributes (i.e., DEM in this experiment) is 0.8. This method has been implemented in GeoDa software. The Ward-like hierarchical clustering algorithm, including spatial/geographical constraints, modifies the measure of dissimilarity between the spatial units in hierarchical clustering. The new dissimilarity is a linear combination of the attribute dissimilarity $D_0$ and the spatial dissimilarity $D_1$. The weight of $D_1$ is $\alpha$, and the weight of $D_0$ is $1-\alpha$. This method is implemented by the R package ClustGeo. Both methods traded off the attribute similarity and location similarity of the clustering results by adjusting the weights. The third category of non-strict spatially constrained clustering did not participate in the comparison due to its requirements for data distribution.

The proposed method provides two different data classification schemes. We used the head/tail breaks scheme for data with heavy-tailed distribution and the natural breaks for data with nonheavy-tailed. The selection of window size, range threshold *R* and standard deviation threshold *V*, and adaptation mechanisms for weak fluctuations can adjust the attribute similarity and location similarity of the clustering results. This paper compares two versions of the new algorithm with the two baseline methods described above.

### Evaluation indicators

We have selected three metrics to evaluate the clustering results. The first metric is the Geodetector q statistic^55^, which measures the spatial stratified heterogeneity. Spatial hierarchical heterogeneity refers to the spatial heterogeneity among strata or regions (each stratum or region consists of several units)^56^, specifically, the geographical phenomenon that the variance within a stratum (i.e., ’cluster’ in this paper) is smaller than the variance between strata. Hence, the higher the requirement of spatial integration in clustering, the less significant the spatial hierarchical heterogeneity of the results.The formulation for the q statistic is

1 $$\begin{aligned} q=1-\frac{\sum _{i=1}^KN_i\sigma _i^2}{N\sigma ^2} \end{aligned}$$

where $i=1,\dots ,K$ are clusters; $N_i$ and *N* are the number of units in cluster *i* and the whole region, respectively; $\sigma _i^2$ and $\sigma$ are the variances in cluster *i* and the whole region, respectively.

The other two metrics are common clustering evaluation indexes, based on the principles of measuring intra-cluster similarity and inter-cluster dissimilarity. These two metrics are the Silhouette Index and the Davies–Bouldin Index. The formula for the Silhouette index of unit x is as follows,

2 $$\begin{aligned} s(x)=\frac{b(x)-a(x)}{max\{a(x),b(x)\}} \end{aligned}$$

where *a*(*x*) is the average of the attribute distances from unit *x* to units of the same cluster, and *b*(*x*) is the minimum value of the average distance from unit *x* to all units in a cluster that does not contain it. The value of the Silhouette Index is between $[-1,1]$, and the closer it is to 1, the better the clustering effect. The formulation for the Davies–Bouldin Index is

3 $$\begin{aligned} \text {DB}=\frac{1}{n} \sum _{i=1}^{n} \max _{j \ne i}(\frac{d_{i}+d_{j}}{d(c_{i}, c_{j})}) \end{aligned}$$

Here, *n* represents the number of clusters, $c_i$ is the center of the i-th cluster, $d_{i}$ is the average distance from all points in cluster i to its center, and $d(c_{i}, c_{j})$ is the distance between the center points $c_{i}$ and $c_{j}$. In the following experiments, we will compare which method obtains a larger Silhouette Index for the same value of *q*. A smaller DB value indicates a better cluster result.

For non-strict spatially constrained clustering, multiple clustering results can be achieved under various spatial-attribute tradeoffs. To ensure comparability between different clustering results, we aim to obtain results with the same q-value using distinct methods and, in this situation, compare the values of the remaining metrics.

## Experimental results

### Results of clustering extreme areas

**Figure 7:**
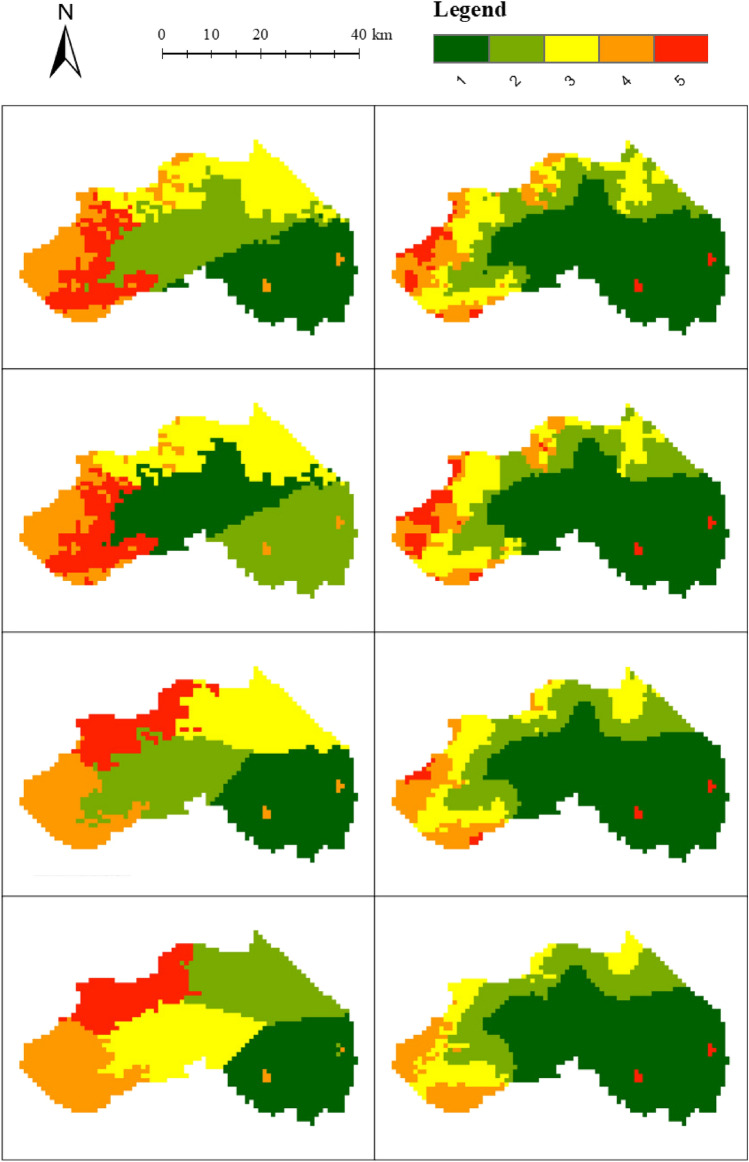
Results of SKM (left side) and NM (right side) for Changping District with extreme areas when *q*s are the same (same row).

The first experiment is carried out on the DEM of Changping District. Since the DEM data in Changping District have a heavy-tailed distribution, the natural breaks scheme was used for data classification. The number of classes is set to 5 (the default number of classes in ArcGIS). As a result, the number of clusters generated by spatial clustering is also 5. The numbers of clusters for SKM and WHS are also set to 5. For SKM and WHS, we set their location weights $\alpha$ to 0.2, 0.4, 0.6 and 0.8, respectively, and calculate the *q* of the spatial clustering results under each weight. The *q*s of the SKM clustering results are 0.908, 0.893, 0.796 and 0.720 in order. For WHS, when the location weight is 0.8, the *q* is too small, and the extreme areas cannot be divided, so no comparison is made. The *q*s of the WHS are 0.836, 0.732 and 0.677, respectively. By adjusting the window size, range threshold, and standard deviation threshold of the proposed method, the *q*s obtained are consistent with the above results. The clustering results of the three methods are shown in Fig. 7 and Supplementary Figure S3.

The robustness to spatial outliers of the proposed method can be seen in the map. For SKM, when the location weight is relatively large (in this experiment the weight is 0.8), it cannot correctly divide the extreme areas. For WHS, the two extreme regions are divided into the same cluster with the northeast. Whereas, according to the DEM values, it is known that their attributes are not similar, which is caused by the location joining the clustering. Instead of improving the spatial integration, this clustering result also weakens the similarity of the attributes. While the proposed method can divide extreme areas in all cases.

The numerical results also show the reasonable clustering of spatial outliers by the proposed method.The metrics of all the clustering results are calculated. The Silhouette Indexes and Davies–Bouldin Indexes corresponding to a *q*s are shown in Supplementary Figure S4 (for simplicity, NM is used to represent the proposed method, the same as below). The figure shows that the Silhouette Indexes of the proposed method are larger than those of the two baseline methods, regardless of the *q*. Additionally, for all values of *q*, the Davies–Bouldin Indexes of the proposed method are greater than that of the two baselines.

**Figure 8:**
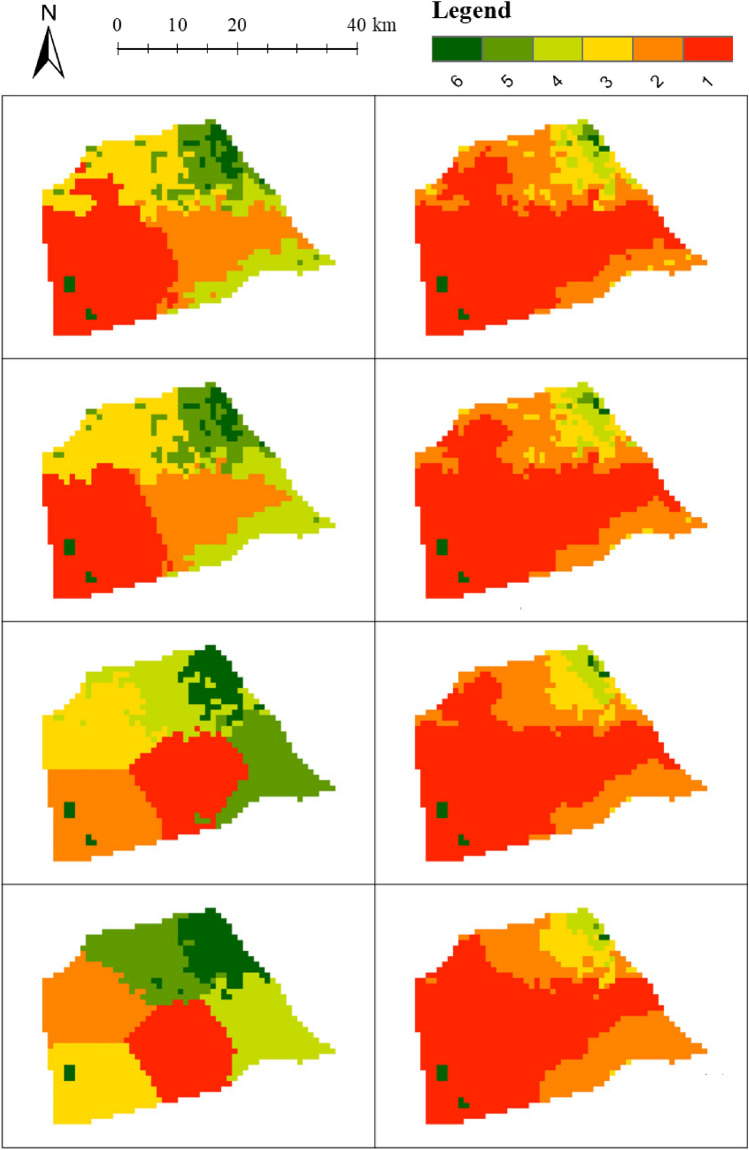
Results of SKM (left side) and NM (right side) for Pinggu District with extreme areas when *q*s are the same (same row).

The following experiment was carried out on the DEM data of Pinggu District. Since the DEM in Pinggu District has a heavy-tailed distribution, the head/tail breaks scheme was used for data classification. The number of classes obtained by the head/tail breaks was 6, and the number of clusters obtained after clustering was 6. The numbers of clusters for SKM and WHS are also set to 5. For SKM and WHS, we set their location weights $\alpha$ to 0.2, 0.4, 0.6 and 0.8, respectively, and calculate the *q* of the spatial clustering results under each weight. The *q*s of the SKM clustering results were 0.919, 0.897, 0.835 and 0.715, respectively. The *q*s of the WHS clustering results are 0.857, 0.816, 0.803 and 0.528 in order. By adjusting the window size, range threshold, and standard deviation threshold of the proposed method, the *q*s obtained are consistent with the above results. The clustering results of the three methods are shown in Figs. 8 and Supplementary Figure S5.

It can be seen from the figures that when the location weight is greater than 0.6, the two baseline algorithms cannot correctly divide the extreme areas.The Silhouette Indexes corresponding to *q*s are shown in Supplementary Figure S6, which shows that the Silhouette Indexes of the proposed method are always larger than those of the two baseline methods. The Davies–Bouldin Indexes of the proposed method are also the best.

### Results of clustering volatility areas

Similarly, the DEM of Changping District is tested first. The natural breaks scheme was also used for data classification. The number of classes is set to 5. Additionally, the numbers of classes generated from SKM and WHS were set to 5. For SKM and WHS, we set their location weights to 0.2, 0.4, 0.6 and 0.8, respectively, and calculated the *q*s of the spatial clustering results under each weight. The *q*s of SKM are 0.912, 0.890, 0.776 and 0.582 in order. The *q*s of the WHS are 0.875, 0.819, 0.817 and 0.623, respectively. By adjusting the window size, range threshold, and standard deviation threshold of the proposed method, the *q*s obtained are consistent with the above results.The clustering results of the three methods are shown in Supplementary Figure S7 and Supplementary Figure S8.

Both baseline algorithms cannot reasonably cluster the volatility areas. When location weight of SKM reaches 0.6, about half of the volatility area merge with the surrounding cluster, and the remaining half are clustered into the same cluster. When the location weight reaches 0.8, the volatility area is completely merged with the surrounding units into one cluster. The result of WHS clusters the volatility area into almost the same cluster at all location weights. And similar to the extreme value region, the volatility area is divided into the same cluster with the northeast. And the proposed method divides the volatility areas to different degrees under various spatial stratified heterogeneity. The Silhouette Indexes shown in Supplementary Figure S9 also reveal that the effect of the proposed method are better. At the same time, in most cases, the Davies–Bouldin Indexes of the proposed method are the best, with only two scenarios where the Davies–Bouldin Indexes of the proposed method are slightly inferior, which correspond to the two worst cases in terms of *q*.

The following experiment was carried out on the DEM data of Pinggu District. The number of classes obtained by the head/tail breaks was 4. Additionally, the numbers of classes generated from SKM and WHS were set to 4. For SKM and WHS, we set their location weights to 0.2, 0.4, 0.6 and 0.8, respectively, and calculated the *q*s of the spatial clustering results under each weight. The *q*s of SKM are 0.890, 0.808, 0.758 and 0.517, respectively. For WHS, when the location weight is 0.8, the *q* is too large, and the volatility areas cannot be divided, so no comparison is made. For WHS, when the location weight is 0.8, the *q* is too small, and the extreme areas cannot be divided, so no comparison is made. The *q*s of the WHS are 0.806, 0.785, 0.527 in order. By adjusting the window size, range threshold, and standard deviation threshold of the proposed method, the *q*s obtained are consistent with the above results. The clustering results of the three methods are shown in Supplementary Figure S10 and Supplementary Figure S11.

Both baseline algorithms are only able to delineate the volatility areas when the location weights are low. However, when the location weight of SKM reaches 0.8, it almost loses the ability to cluster volatility area, while when the location weight of WHS reaches 0.6, the volatility area are divided into the same cluster with Northwest which is closer rather than with more similar attributes. The Silhouette Indexes and Davies–Bouldin Indexes of the proposed method shown in Supplementary Figure S12 are also better than two baseline algorithms.

## Discussion

Regarding the experimental results, we note that the clustering evaluation indexes of the results obtained by the two baseline methods is always worse than that of the proposed method. On the one hand, as previously analysed, the proposed method can better delineate extreme and volatile regions. On the other hand, these two baseline methods tend to divide similar spatial units with similar attributes into different clusters, which is also one of the reasons for the poor performance of the clustering evaluation indexes. The results showed that although non-strict spatially constrained clustering relaxes the enforcement of spatial constraints when compared with strict spatially constrained clustering, the result that similar regions are separated sometimes cannot be avoided. This may be due to the fact that the inclusion of location makes the algorithm tend to generate ’circular’ clusters, which have the highest similarity in the location of spatial units. In contrast, the shape of the cluster generated by the proposed method is only related to the characteristics of the data itself, and there is no tendency to cluster into a certain shape.

During the clustering process, it is worth noting that the adaptation mechanisms indirectly affect the clustering results. The two adaptation mechanisms have different advantages. From the numerical results, the averaging of *q* is better than the occupying of *q*. It is easy to understand because averaging turns the grids in the window into a compromised cluster so that the attribute values of the grids do not differ too much from that cluster. As a result, this method is mathematically optimal. However, occupying expands the area of the cluster in the local area and optimizes the spatial structure of the clusters. Therefore, when performing spatial clustering, averaging is more suitable if it focuses on the mathematical optimization; if it focuses on the optimization of the spatial structure, occupying is more suitable.

The influence of window size on clustering results is also a worthwhile topic to explore. To compare the effects of different window sizes, we designed an experiment. While keeping the range threshold and standard deviation threshold constant, we compared the clustering results under different window sizes. We conducted experiments on the Changping District DEM data with extreme areas and volatility area, respectively. Figures S13 depicst the spatial distribution of clustering results. It can be observed that the spatial contiguity of the clustering results initially increases and then decreases. As seen on Figures S13, when the window size is between 4 and 6, spatial continuity is at its highest, and subsequently, clustering results gradually become more fragmented. This is because with a smaller window size, increasing it results in an increase in the number of grids that need to change their categories. However, as the window size continues to increase, the number of grids covered by the sliding window also increases, potentially leading to higher range and standard deviation values. With the range and standard deviation thresholds held constant, the number of grids with changed classes decreases, resulting in a more fragmented spatial outcome. Therefore, in practical usage, window size, range threshold, and standard deviation threshold need to be carefully coordinated to achieve the desired clustering effect.

## Conclusion

This study proposes a non-strict spatial clustering method for univariate raster data. This method uses data classification schemes to classify the research area initially and then obtains each locality of the research area through a sliding window. For all grids in the sliding window, the spatial outliers are delineated by judging their attribute values’ range and standard deviation. For windows with a range and standard deviation that are too large, the classes of the initial classification are retained to preserve the spatial outliers. Otherwise, the grids with relatively close distances are classified into one cluster to improve the spatial integration. To prove the effectiveness of the proposed method, we designed a comparative experiment involving the proposed method and two other representative methods. The research areas were selected to facilitate the comparison by including two types of outlier areas, namely, extreme areas and volatility areas. The results demonstrated the advantage of the proposed method; it kept outlier areas in the large-scale contiguous clusters and avoided the problem of similar units being divided into different clusters, whereas the other representative methods did not. Because the proposed method involves the specification of several parameters, pre-experiment debugging is usually required to obtain clustering results that meet users’ needs. In future research, we can try to establish the correlation between parameter changes and clustering results to improve the efficiency of parameter setting. By extending the definitions of the range and standard deviation to higher dimensions, the proposed method can be applied to multivariate data.

### Supplementary Information

Supplementary Information.

## Data Availability

The DEM data used in this study can be obtained from the Resource and Environment Science and Data Center (https://www.resdc.cn/data.aspx?DATAID=123).
