# Supplementary material for: A raster-based spatial clustering method with robustness to spatial outliers
Source: Sci Rep. 2024 Feb 19;14:4103. doi: 10.1038/s41598-024-53066-4 (PMC10876529; doi:10.1038/s41598-024-53066-4)
Supplement: Supplementary file 1 — Supplementary Information. [file 41598_2024_53066_MOESM1_ESM.docx]

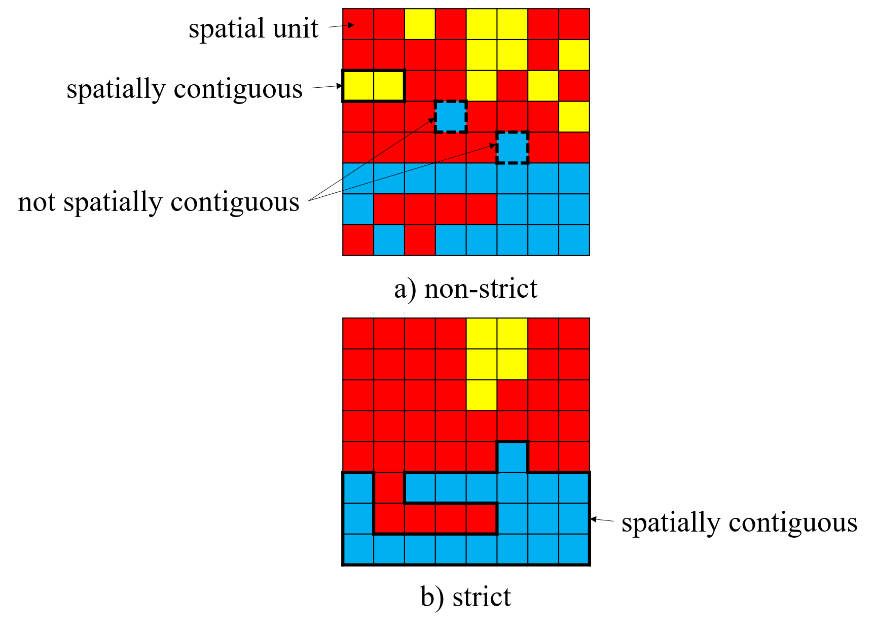


Supplementary Figure S1. Results of the non-strict and strict constraints


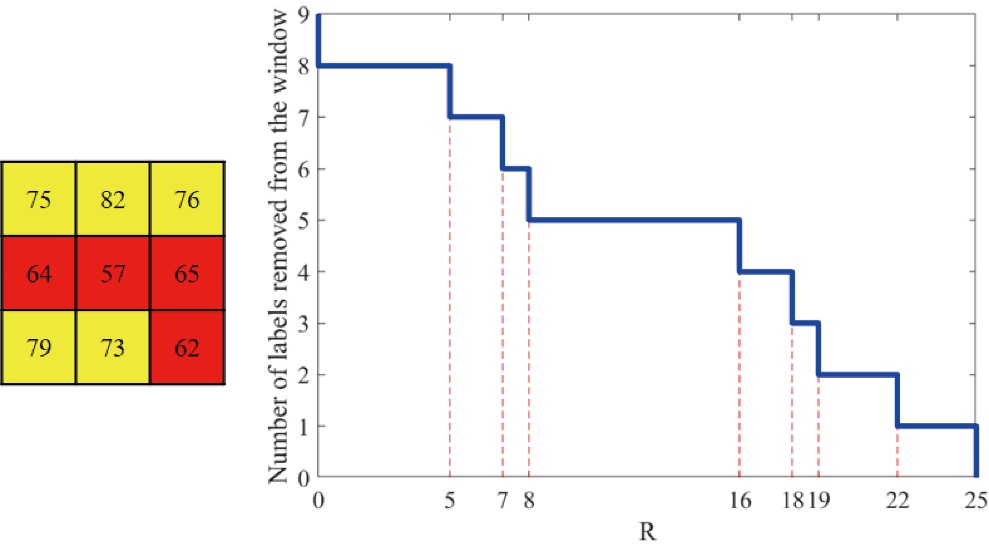


Supplementary Figure S2. The relationship between the number of removed grids and the range threshold R in the left sliding window


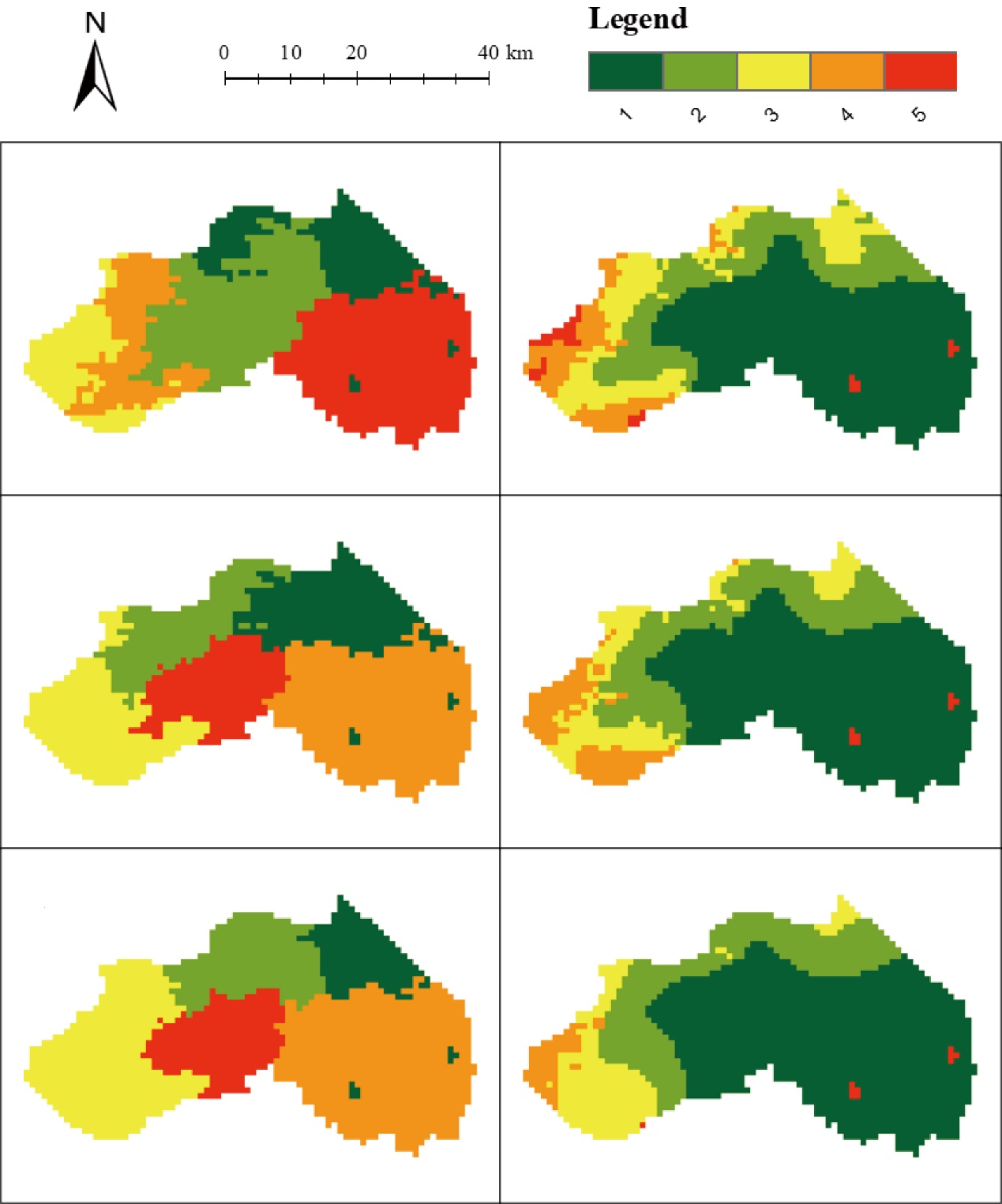


Supplementary Figure S3. Results of WHS (left side) and NM (right side) for Changping District with extreme areas when qs are the same (same row)


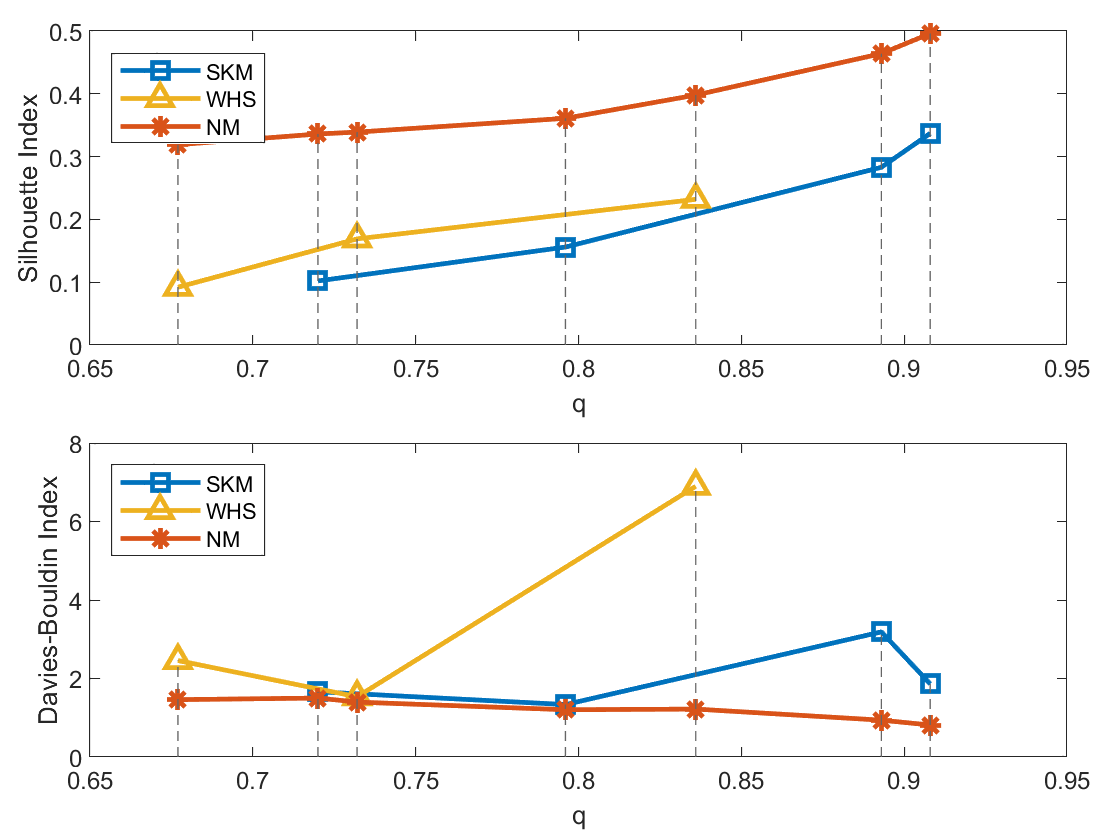


Supplementary Figure S4. Metrics of the proposed method and two baseline methods

for Changping District with extreme areas


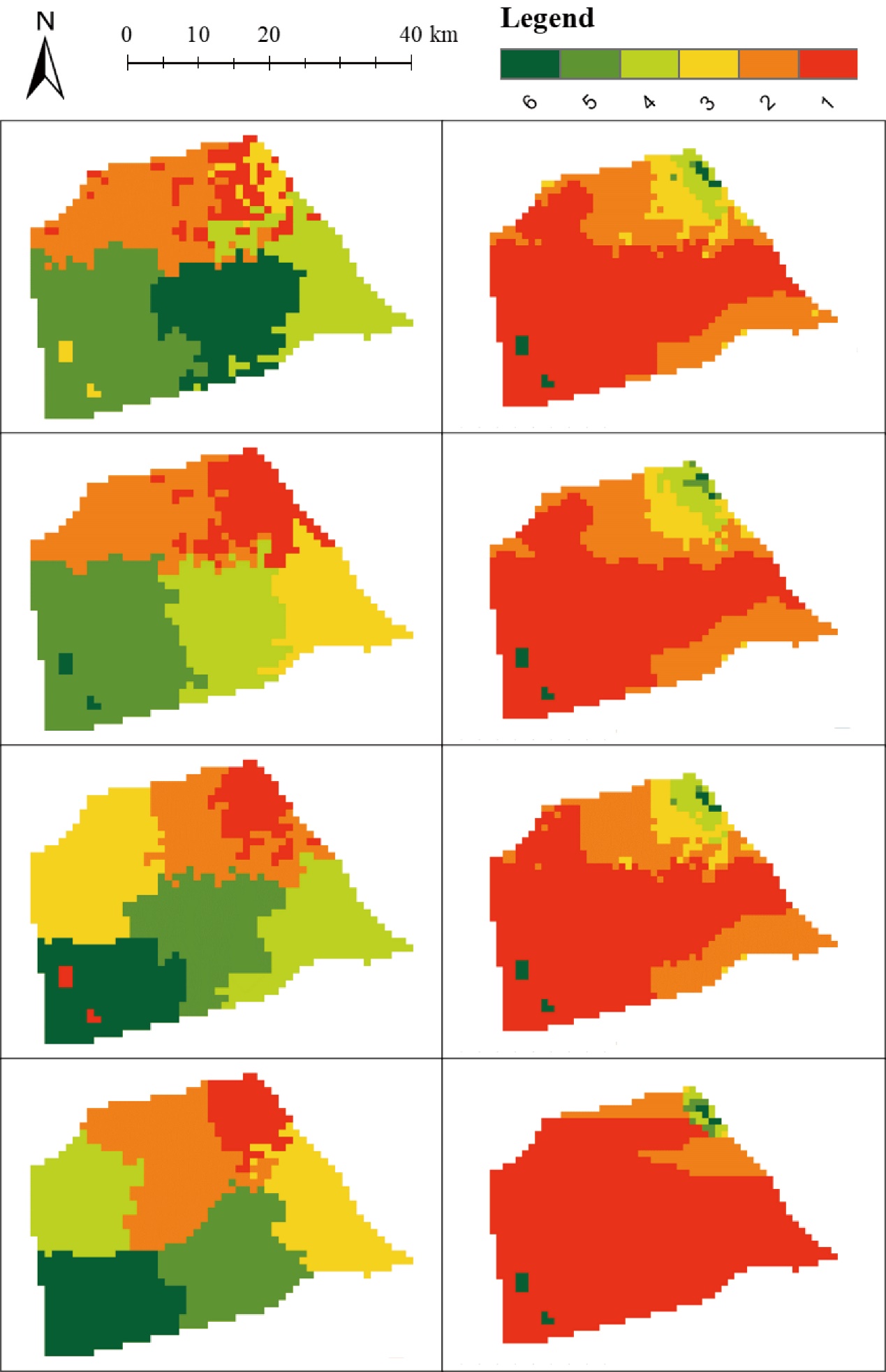


Supplementary Figure S5. Results of WHS (left side) and NM (right side) for Pinggu District with extreme areas when qs are the same (same row)


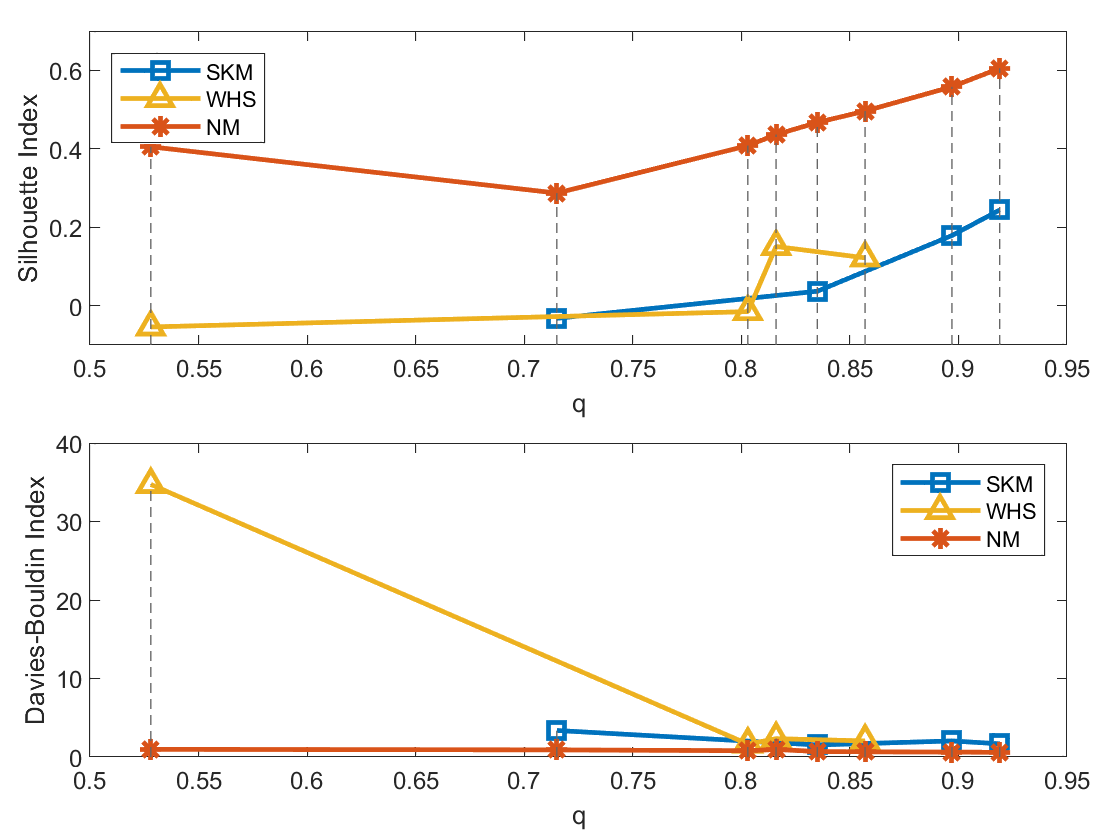


Supplementary Figure S6. Metrics of the proposed method and two baseline methods for Pinggu District with extreme areas


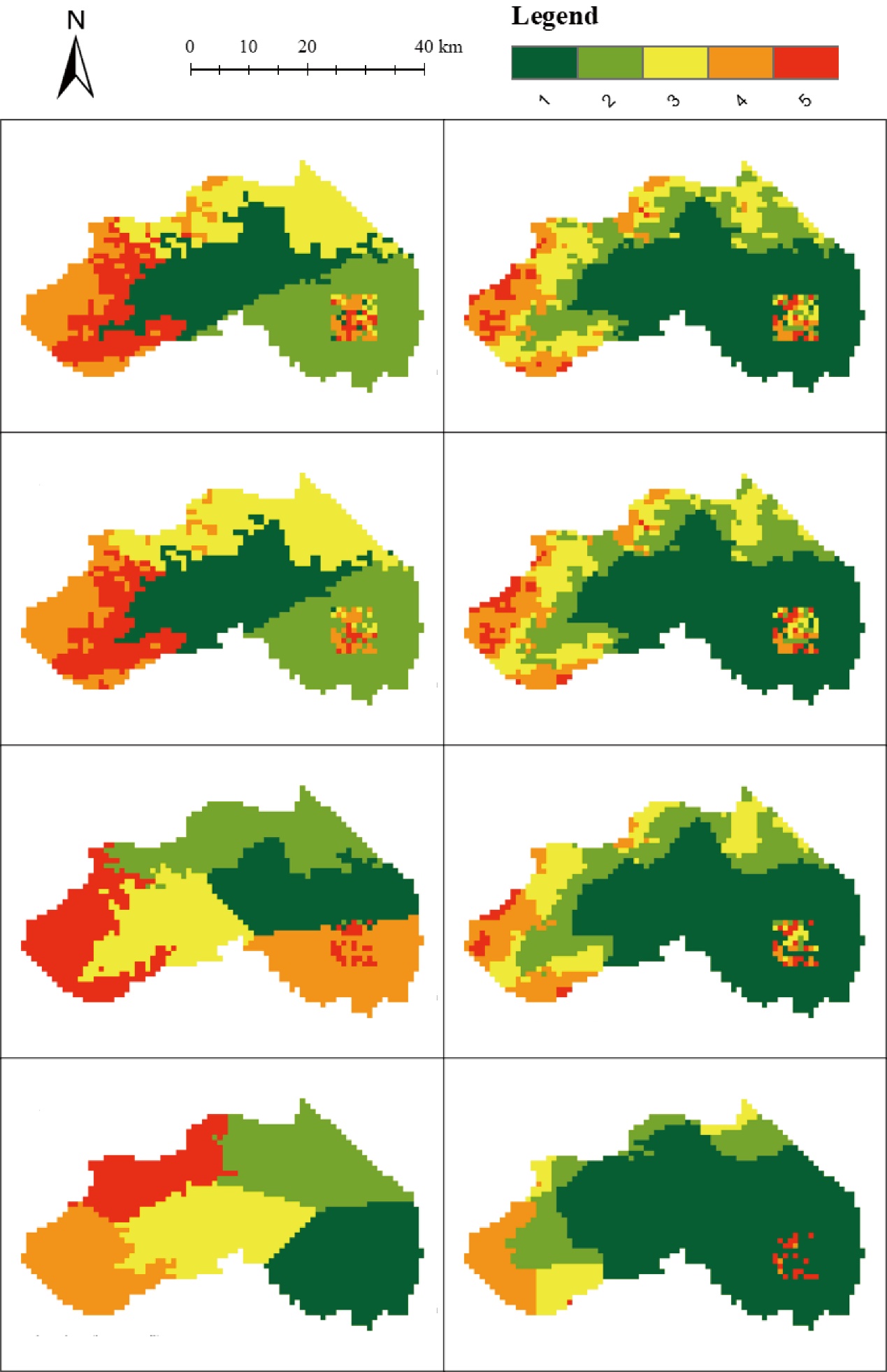


Supplementary Figure S7. Results of SKM (left side) and NM (right side) for Changping District with volatility areas when qs are the same (same row)


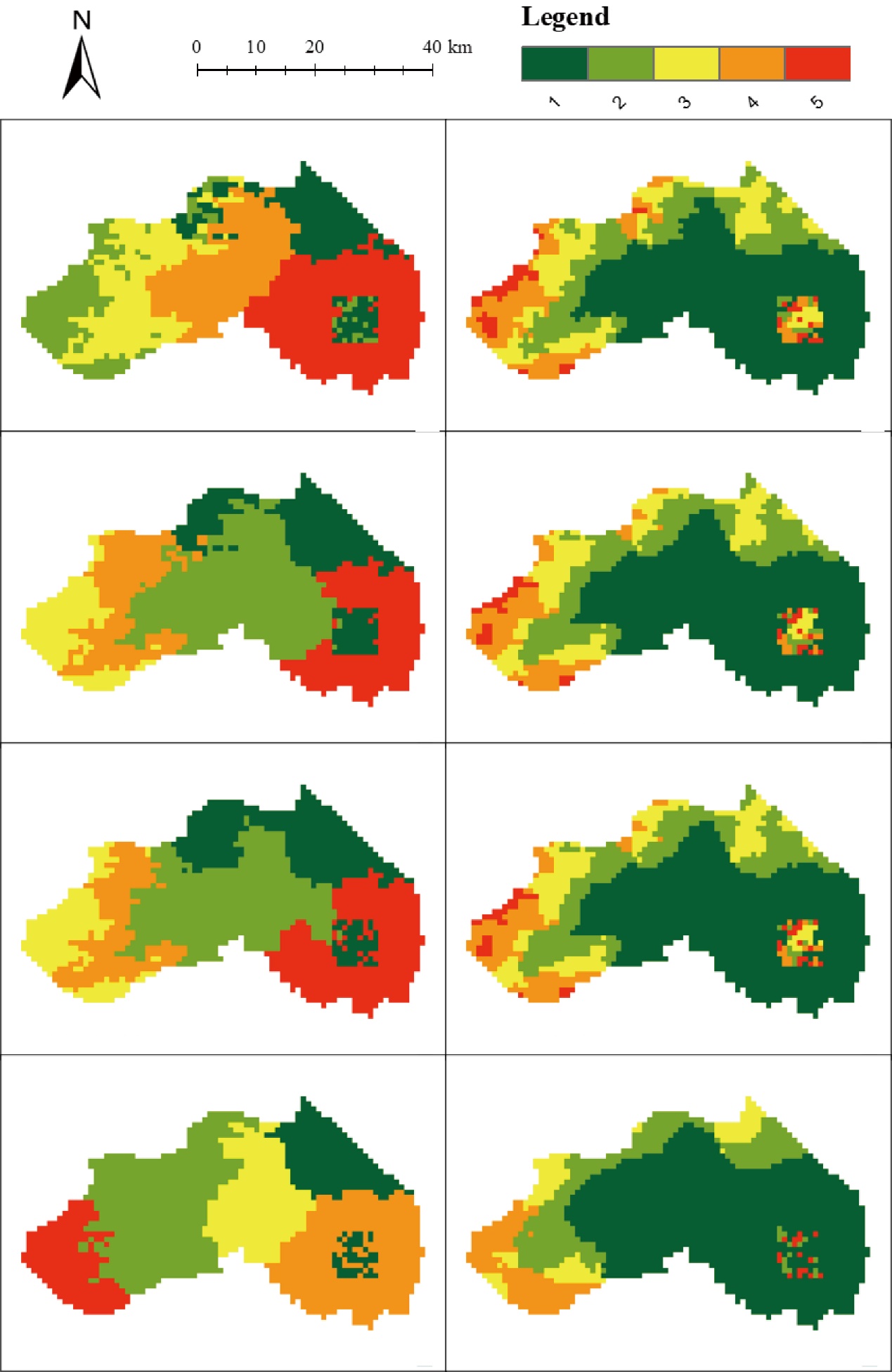


Supplementary Figure S8 Results of WHS (left side) and NM (right side) for Changping District with volatility areas when qs are the same (same row)


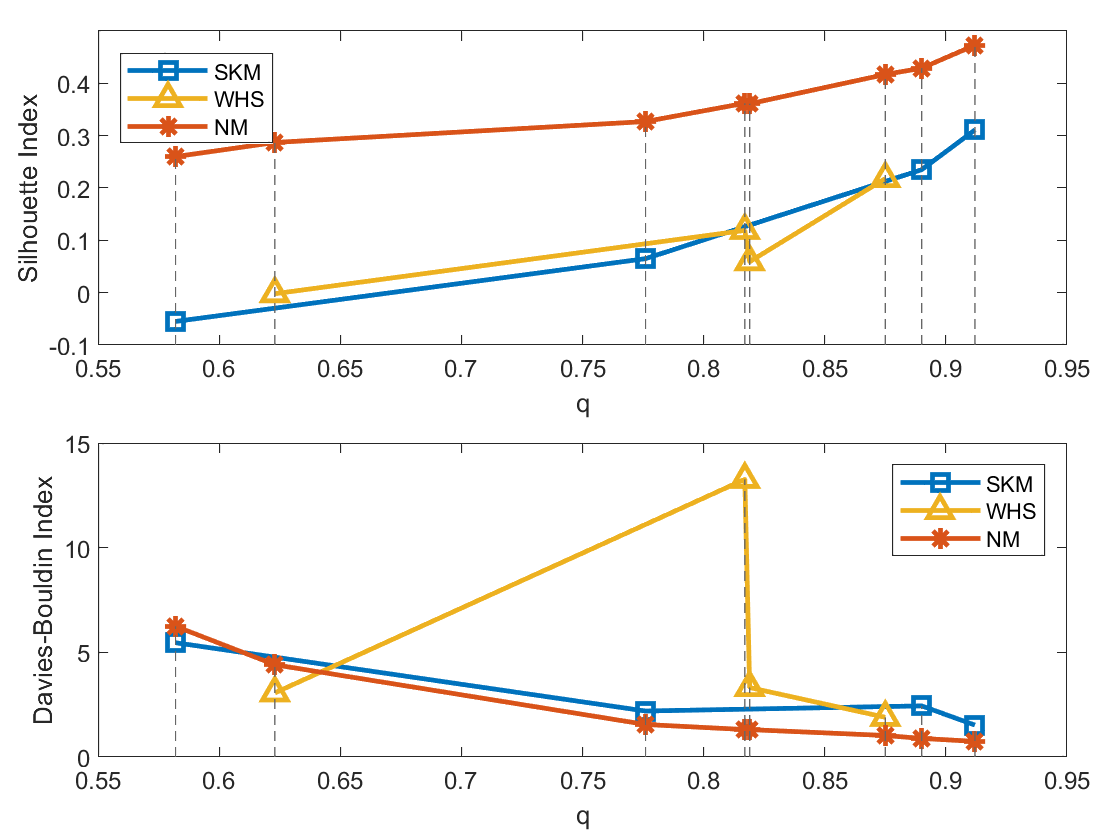


Supplementary Figure S9. Metrics of the proposed method and two baseline methods for Changping District with volatility areas


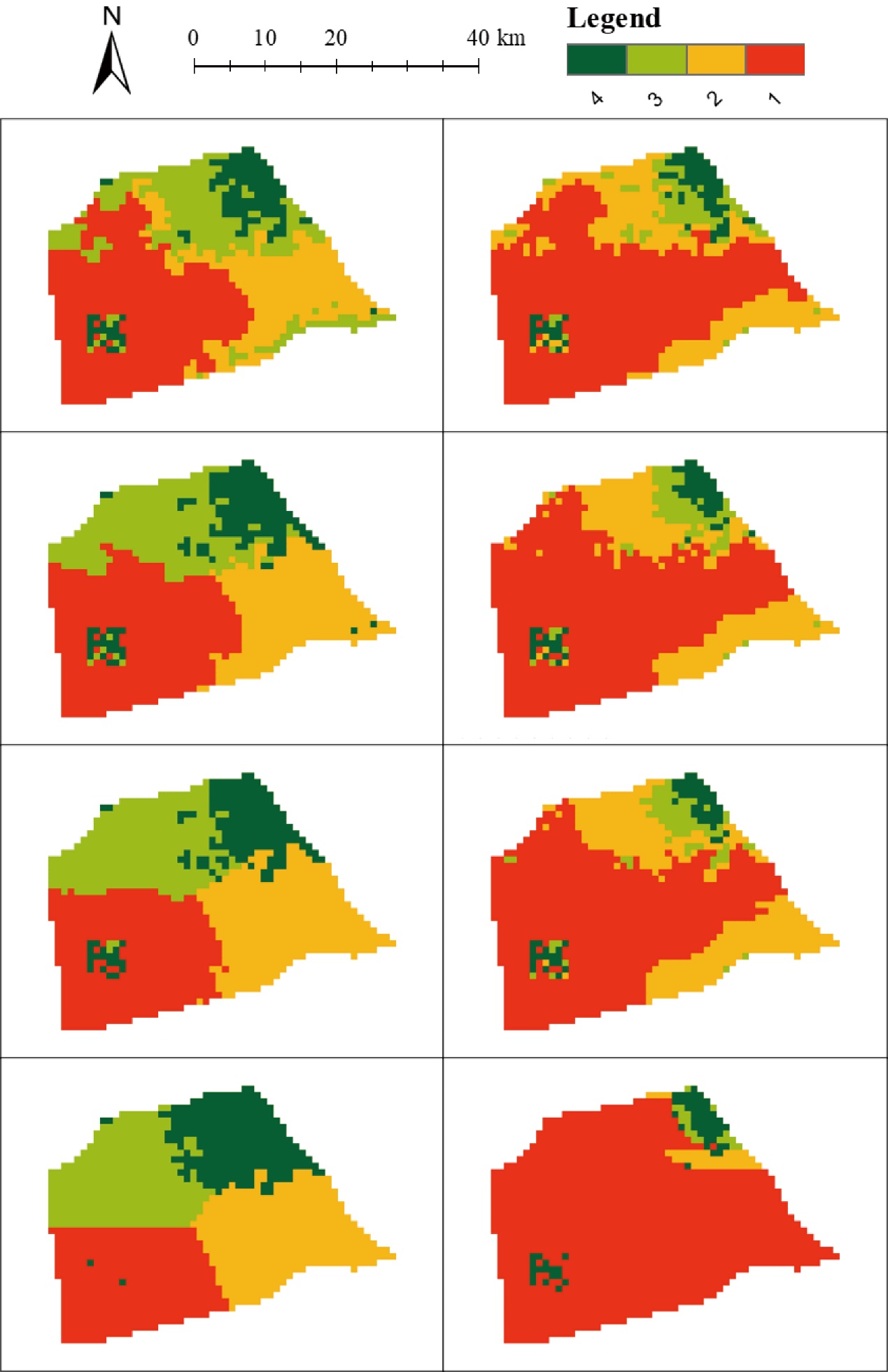


Supplementary Figure S10. Results of SKM (left side) and NM (right side) for Pinggu District with volatility areas when qs are the same (same row)


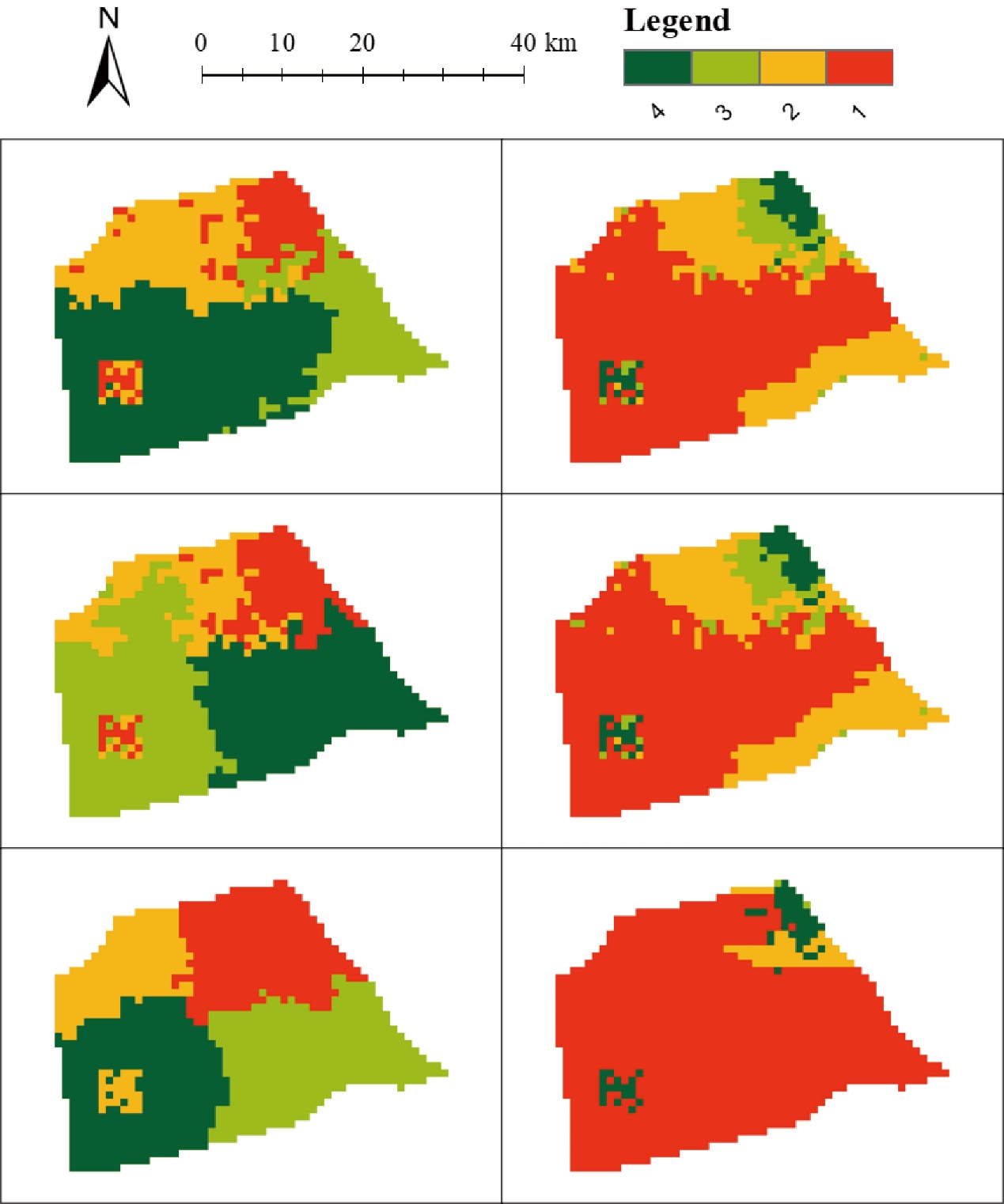


Supplementary Figure S11. Results of WHS (left side) and NM (right side) for Pinggu District with volatility areas when qs are the same (same row)


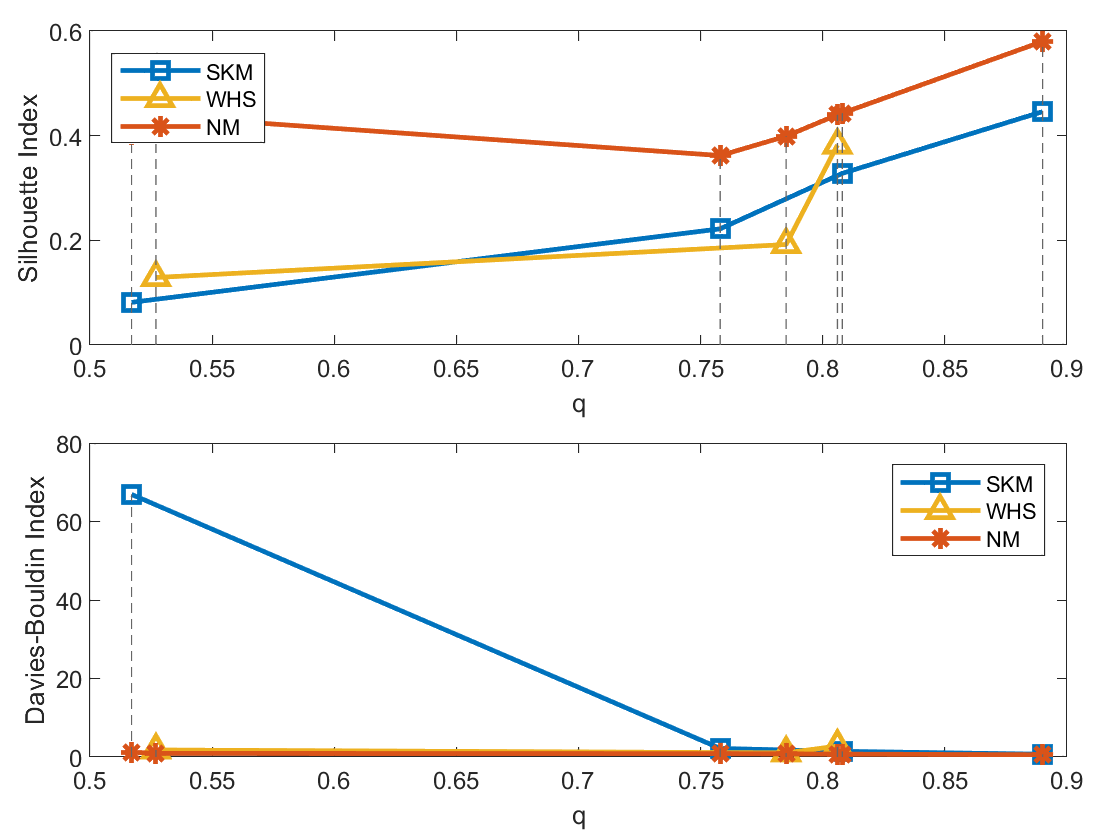


Supplementary Figure S12. Metrics of the proposed method and two baseline methods for Pinggu District with volatility areas


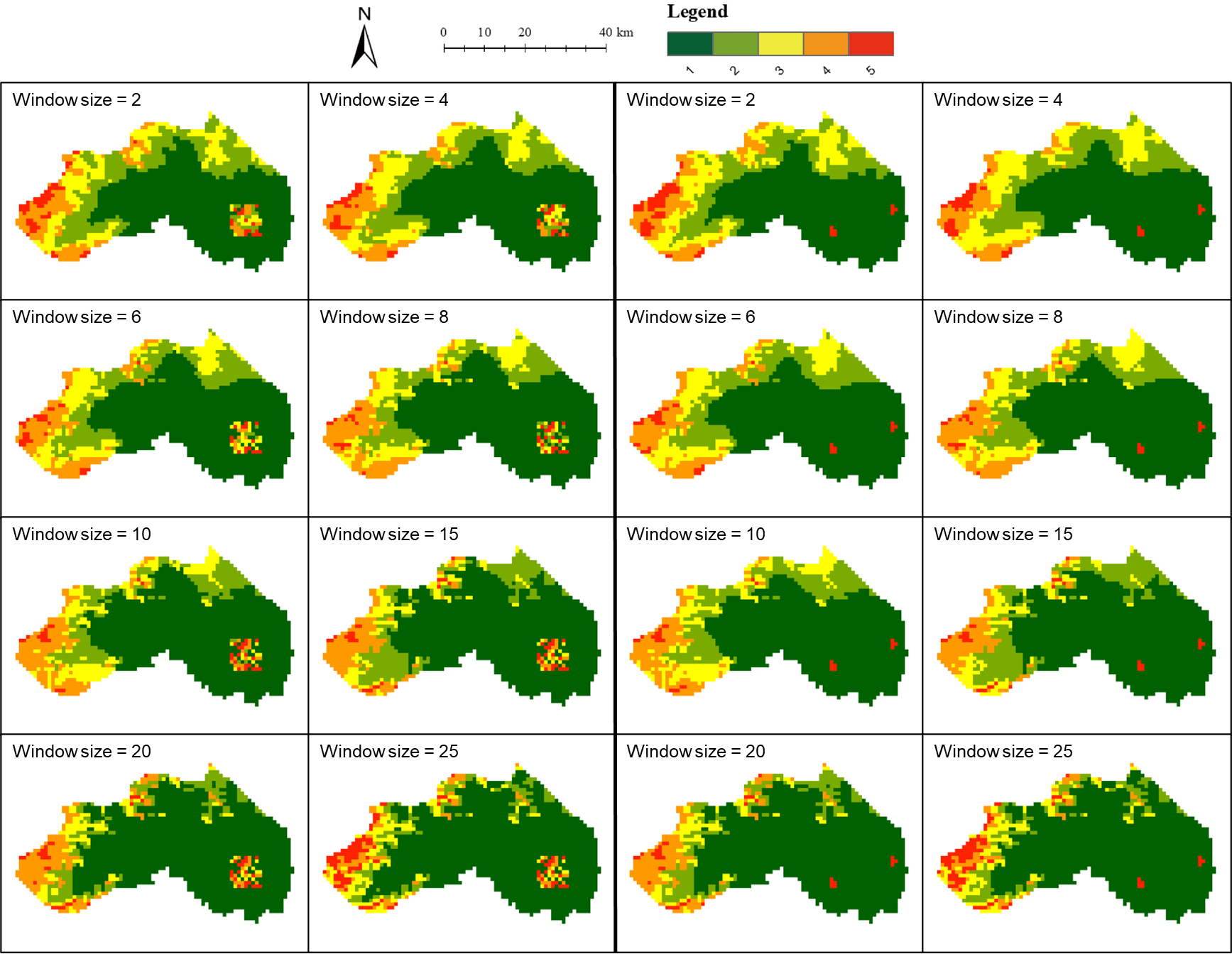


Supplementary Figure 13 Clustering results under different window sizes (with the same range threshold and standard deviation threshold).
